# Supplementary material for: Sustained-input switches for transcription factors and microRNAs are central building blocks of eukaryotic gene circuits
Source: Genome Biol. 2013 Aug 23;14(8):R85. doi: 10.1186/gb-2013-14-8-r85 (PMC4054853; doi:10.1186/gb-2013-14-8-r85)
Supplement: Additional file 5 — HTML Browsable Motif Output. Zipped folder containing all WaRSwap and FANMOD motif output, viewable in a web browser. [file gb-2013-14-8-r85-S5.ZIP › HTML_browsable_motif_output/WaRSwap_ath_tair9/sigs_WSWRSLDraw_2-2000.pvals.heatmaps.html/motif_id_38_000101101_tftype_ath_upstream_-3000_0.html]

```
BG_MODEL = Rnew2
MOTIF_ID = 38_000101101
TF_TYPE = ath
UPSTREAM = -3000_0


PVals
FN_0.2	FN_0.4	FN_0.6	FN_0.8
dg_60.genes	0.167	0.246	0.028	0.021
dg_70.genes	0.233	0.281	0.018	0.024
dg_80.genes	0.105	0.116	0.007	0.016

ZScores
FN_0.2	FN_0.4	FN_0.6	FN_0.8
dg_60.genes	1.02	0.693	2.06	2.605
dg_70.genes	0.804	0.595	2.367	2.729
dg_80.genes	1.383	1.285	2.887	2.924

StDevs
FN_0.2	FN_0.4	FN_0.6	FN_0.8
dg_60.genes	61.232	33.483	13.329	4.414
dg_70.genes	60.627	33.305	12.286	4.041
dg_80.genes	53.931	29.576	11.596	3.965
```
